# Criticism by community people and poor workplace communication as risk factors for the mental health of local welfare workers after the Great East Japan Earthquake: A cross-sectional study

**DOI:** 10.1371/journal.pone.0185930

**Published:** 2017-11-22

**Authors:** Ikki Ueda, Atsushi Sakuma, Yoko Takahashi, Wataru Shoji, Ayami Nagao, Mikika Abe, Yuriko Suzuki, Hiroo Matsuoka, Kazunori Matsumoto

**Affiliations:** 1 Department of Psychiatry, Tohoku University Graduate School of Medicine, Sendai, Miyagi, Japan; 2 Miyagi Disaster Mental Health Care Center, Sendai, Miyagi, Japan; 3 Department of Psychiatry, Tohoku University Hospital, Sendai, Miyagi, Japan; 4 Department of Preventive Psychiatry, Tohoku University Graduate School of Medicine, Sendai, Miyagi, Japan; 5 Department of Adult Mental Health, National Institute of Mental Health, National Center of Neurology and Psychiatry, Tokyo, Japan; National Defense Medical College Research Institute, JAPAN

## Abstract

After a large-scale natural disaster, demand for social welfare services increases, and the mental health of local social welfare workers becomes a matter of great concern because of their dual role as support providers and disaster survivors. We examined whether work-related social stressors, including criticism by community people and poor workplace communication, were associated with increased risk of post-traumatic stress disorder (PTSD), depression, or psychological distress 20–22 months after the Great East Japan Earthquake (GEJE; March 11, 2011) in local social welfare workers. Demographic characteristics, disaster-related risk factors (near-death experience, dead/missing family members, loss of housing), and work-related social risk factors (criticism, lack of communication) were obtained 20–22 months after the GEJE from 822 local workers. Questionnaires measured PTSD, depression, and psychological stress. Bivariate and multivariate regression analyses were applied. More local social welfare workers suffered from mental health problems than would be expected. Criticism by community people was significantly associated with probable PTSD and high psychological distress (adjusted odds ratio = 2.31 and 2.55, respectively). Furthermore, lack of workplace communication was associated with probable PTSD, depression, and high psychological distress (adjusted odds ratio = 3.97, 4.27, and 4.65, respectively). Almost 2 years after the disaster, local relief workers still suffered from mental health problems. Because post-disaster work-related social stressors constitute risk factors for these mental health problems, measures to improve working conditions and prevent and treat mental disorders should be a priority.

## Introduction

Various occupations are known to be involved in the long-term recovery process after large-scale disasters, and many studies have focused on the psychological sequelae of disaster rescue workers or “traditional” first responders [1]. However, studies have recently begun to focus on the mental health of local workers who are involved in the recovery process of the affected community and are also disaster survivors, such as municipality workers [2–4], medical workers [2,5], or disaster volunteers [6]. These local workers play an indispensable role in reconstructing devastated communities and are often involved in relief efforts and the reconstruction of affected areas long after the immediate aftermath of a disaster. It is known that these local workers are at increased risk of developing post-traumatic stress disorder (PTSD) and depression due to direct and indirect causes resulting from large-scale disasters [2].

On March 11, 2011, one of the largest earthquakes ever recorded in Japan occurred off the coast of Japan (Great East Japan Earthquake, GEJE). This earthquake had a magnitude of 9.0 on the Richter scale. It hit the northern and eastern coasts of Japan in the Tohoku region. The Miyagi prefecture was closest to the epicenter of the earthquake and was the most affected area. Immediately after the earthquake, a large tsunami struck nearly all of the coastal cities and towns of the Miyagi prefecture, which were severely damaged. This resulted in approximately 400,000 houses being destroyed, and 18,465 people being killed or going missing within the entire country. In the Miyagi prefecture, approximately 240,000 houses were destroyed, and 10,780 people were killed or went missing [7]. As always with large-scale natural disasters, the GEJE wreaked enormous damage on entire communities with many residents suffering the loss of family members or friends, possessions, jobs, homes, and health.

After such a massive disaster, many people suddenly become socially vulnerable and require support to protect their livelihood and restore a sense of connectedness in the community. Elderly persons, people with disabilities, pregnant women, and single parent families are among the people affected who were at greater risk. They require special attention and care during and after the disaster, in some cases for lengthy periods.

Thus, the demands for social welfare increase after a large-scale disaster [8,9]. To compensate for such increased demands, many supporters including professionals as well as non-professional volunteers outside of the disaster-affected area come to be involved in disaster relief activities mainly during the early phase of the disaster. However, those supporters from outside of the area decrease over time because of the limited role of outside supporters during the later phase of the disaster. Meanwhile, local welfare workers who reside in the disaster-affected area are continuously involved in relief and reconstruction long after the immediate aftermath of a disaster. The Japan Councils of Social Welfare (SHAKYOs), which are non-profit private organizations, perform a central role in disaster assistance in the social welfare domain in Japan. Based on the Social Welfare Act, the SHAKYOs are established within the administrative units of local governments (i.e., in every prefecture, city, town, and village). In ordinary times, the local SHAKYOs coordinate among governmental organizations (e.g., local government, public health centers, child consultation centers) and related organizations (e.g., social welfare corporations, the Welfare Volunteers and the Child Welfare Volunteers, voluntary groups, non-profit and non-governmental organizations) to develop welfare projects in response to the communities’ needs. Additionally, once a large-scale disaster happens, the SHAKYOs take the role of comprehensive coordinators for welfare assistance in the affected communities, ensuring the safety of vulnerable people needing special evacuation assistance from their homes and institutions, providing temporary evacuation shelters, running welfare shelters, assisting ordinary shelters, setting up and running disaster volunteer centers, providing loans under the welfare system, and providing assistance to people living in temporary housing.

SHAKYO workers are also residents of the disaster-affected area, that is, survivors of the disaster. Because they directly support the survivors in face-to-face relationships, they frequently experience a role conflict between survivor and support provider. Additionally, although the need for social welfare services increases after a large-scale natural disaster [8,9], social resources of the disaster-affected area are generally limited, and it is sometimes difficult to supply sufficient social welfare services in response to increased demand. Accordingly, they are sometimes criticized by community people including not only their service users, but also other survivors or people with complaints about insufficient supply of services in the community.

Because such disaster-related personal and workplace factors are expected to affect the mental health of local workers [2,3], their mental health status has been a matter of great concern.

### Aims of the study

In this study, we investigated the mental health conditions of local welfare workers who belonged to the local SHAKYOs in the coastal area of the Miyagi prefecture 20–22 months after the GEJE. We hypothesized that many local workers suffered from mental health problems such as post-traumatic stress disorder (PTSD), depression, and/or psychological distress. We also hypothesized that factors related to the disaster itself and workplace factors, including criticism by community people and poor social communications were associated with increased risk for developing these mental health problems.

## Materials and methods

### Participants

In this study, mental health conditions and related factors were assessed among the workers of local SHAKYOs in the tsunami-affected area of the GEJE. The study was conducted from November 2012 to January 2013 (i.e., 20–22 months after the GEJE) using self-administered questionnaires.

The study used data from workplace health examinations of local SHAKYOs organized by the Miyagi Prefecture SHAKYO and the Department of Preventive Psychiatry, Tohoku University Graduate School of Medicine. Six local SHAKYOs of Kesennuma city, Minamisanriku town, Ishinomaki city, Onagawa town, Shichigahama town, and Sendai city participated in the survey. The coastal areas of these municipalities were among the hardest hit by the earthquake and tsunami. The survey data were also used for planning mental health support programs in each SHAKYO.

All workers belonging to the 6 local SHAKYOs (n = 1008) were invited to participate in the study. Then, the questionnaires were distributed to 1008 workers, and 822 (81.5%) returned them. We included 819 (81.3%) workers who responded to enough questions to screen for probable PTSD, probable depression, and high general psychological distress. We could not access the information of those who did not participate in the study.

### Assessment

Self-administered questionnaires were used to gather data on demographic characteristics (age, gender, educational qualification, pre-existing medical history, psychiatric treatment history, living alone or not, nature of house damage, and working at the SHAKYOs before or after the GEJE), disaster-related personal factors, and workplace risk factors (coded dichotomously as “yes” or “no”). We assessed whether our subjects had suffered near-death experiences, dead or missing family member(s), and displacement as disaster-related personal factors, and whether they had experienced criticism from community people (i.e., not only their service users, but also other survivors or people with complaints about their services), lack of rest, and lack of communication as workplace factors, as described in detail previously [2].

PTSD symptoms, depressive symptoms, and general psychological distress were assessed with the PTSD Checklist–Specific Version (PCL-S) [10], the Patient Health Questionnaire-9 (PHQ-9) [11], and the K6 scale [12], respectively, as described in detail previously [2]. We used a total cutoff score of 44 [13] on the PCL-S as the definition of probable PTSD, a total cutoff score of 10 [14] on the PHQ-9 as the definition of probable depression, and a total cutoff score of 13 [15,16] as having a high level of general psychological distress.

### Statistical analysis

Descriptive analyses for demographic characteristics, prevalence rates for probable PTSD, probable depression, and high general psychological distress were conducted using SPSS version 20.0 (SPSS Inc., Chicago, Illinois). Two-tailed χ^2^ tests complemented by adjusted residual analysis were used to evaluate differences in categorical variables, and one-way analyses of variance were used to analyze continuous variables.

Significant independent variables from the bivariate analysis were considered potential factors for high general psychological distress, probable PTSD, and probable depression and were entered into the multivariate logistic regression model (forced-entry method), as reported previously [2,17]. A two-sided P < 0.05 was used to indicate significance.

### Ethical issues

The data used in this study were acquired during health examinations conducted at each workplace of the 6 local SHAKYOs. To protect the privacy of participants, the questionnaires were distributed and collected within each workplace by the person who oversaw the health of staff members. We obtained the electronic data but not the personal information. Therefore, we could not obtain written informed consent from each participant. Instead, we disclosed the study information, including the objectives and procedure, to the subjects and provided them with the opportunity to refuse participation. All participants who completed and returned the questionnaires were deemed to consent to the study. The rights and welfare of participants were protected as per the ethical guidelines of the Declaration of Helsinki, and the ethical principles of the Ministry of Health, Labour and Welfare of Japan were upheld. The study protocol and consent procedure were reviewed and approved by the Ethics Committee of Tohoku University Graduate School of Medicine (reference number: 2012-1-197).

## Results

### Demographic characteristics and prevalence of mental health problems

Table 1 shows the demographic characteristics, and prevalence of probable PTSD, probable depression, and high general psychological distress.

**Table 1:** Participant characteristics and psychological symptoms.

	Total N = 819		(95%Cl)
**Age (mean (SD))**	46.7	(10.7)	(46.0–47.5)
**Gender**			
Male	219	26.7	(23.7–30.0)
Female	587	71.7	(68.6–74.8)
Unknown	13	1.6	(0.7–2.4)
**Educational qualification**			
Middle school	26	3.2	(2.0–4.4)
High school	375	45.8	(42.4–49.2)
Vocational school	171	20.9	(18.1–23.7)
Junior college	66	8.1	(6.2–9.9)
Bachelor’s degree or higher	175	21.4	(18.6–24.2)
Unknown	6	0.7	(0.1–1.3)
**Preexisting illnesses**			
Yes	262	32.0	(28.8–35.2)
No	550	67.2	(63.9–70.4)
Unknown	7	0.9	(0.2–1.5)
**Psychiatric treatment history**			
Yes	54	6.6	(4.9–8.3)
No	753	91.9	(90.1–93.8)
Unknown	12	1.5	(0.6–2.3)
**Living alone**			
Yes	50	6.1	(4.5–7.7)
No	758	92.6	(90.8–94.4)
Unknown	11	1.3	(0.6–2.1)
**Nature of house damage**			
Total collapse	210	25.6	(22.7–28.6)
Massive collapse	41	5.0	(3.5–6.5)
Half collapse	60	7.3	(5.5–9.1)
Partial collapse	222	27.1	(24.1–30.2)
Almost no damage or none	282	34.4	(31.2–37.7)
Unknown	4	0.5	(0.0–1.0)
**Working at SHAKYOs before GEJE**			
Yes	491	60.0	(56.6–63.3)
No	302	36.9	(33.6–40.2)
Unknown	26	3.2	(2.0–4.4)
**Disaster-related personal factors**			
Near-death experience			
Yes	529	64.6	(61.3–67.9)
No	275	33.6	(30.3–36.8)
Unknown	15	1.8	(0.9–2.7)
Dead or missing family member(s)			
Yes	68	8.3	(6.4–10.2)
No	744	90.8	(88.9–92.8)
Unknown	7	0.9	(0.2–1.5)
Displacement			
Yes	217	26.5	(23.5–29.5)
No	583	71.2	(68.1–74.3)
Unknown	19	2.3	(1.3–3.4)
**Workplace factors**			
Criticism by community people			
Yes	242	29.5	(26.4–32.7)
No	569	69.5	(66.3–72.6)
Unknown	8	1.0	(0.3–1.7)
Lack of rest			
Yes	305	37.2	(33.9–40.6)
No	506	61.8	(58.5–65.1)
Unknown	8	1.0	(0.3–1.7)
Lack of communication			
Yes	302	36.9	(33.6–40.2)
No	511	62.4	(59.1–65.7)
Unknown	6	0.7	(0.1–1.3)
**Psychological symptoms**			
Probable PTSD			
PCL score ≧ 44	33	4.0	(2.7–5.4)
PCL score < 44	770	94.0	(92.4–95.6)
Unknown	16	2.0	(1.0–2.9)
Probable depression			
PHQ-9 score ≧ 10	101	12.3	(10.1–14.6)
PHQ-9 score < 10	676	82.5	(79.9–85.1)
Unknown	42	5.1	(3.6–6.6)
High general psychological distress			
K6 score ≧ 13	65	7.9	(6.1–9.8)
K6 score < 13	718	87.7	(85.4–89.9)
Unknown	36	4.4	(3.0–5.8)

SD = standard deviation, CI = confidence interval

A total of 733 (89.5%) participants completed all 3 measures, 78 (9.5%) completed 2 measures, and 8 (1.0%) completed only 1 measure. Demographic characteristics and experience of risk factors did not differ according to the number of completed measures. Because most participants were residents of the affected area, many of them had severe disaster-related experiences. In total, 529 (64.6%) workers reported a “near-death experience,” and 217 (26.5%) workers were displaced from their homes because of the damage caused by the GEJE. Among the workplace factors, 305 (37.2%) workers reported “lack of rest,” 302 (36.9%) reported “lack of communication,” and 242 (29.5%) workers felt hurt by “criticism from community people.” The prevalence of probable PTSD, probable depression, and high general psychological distress was 4.0%, 12.3%, and 7.9%, respectively.

Among participants who completed all 3 measures, 614 (83.8%) did not meet any of the 3 high-risk criteria, 65 (8.9%) met 1 criterion (i.e., 5 met a PCL criterion, 44 met a depression criterion, and 16 met a K6 criterion), 42 (5.7%) met 2 criteria (i.e., 12 met PCL and PHQ-9 criteria, 28 met PHQ-9 and K6 criteria, and 2 met PCL and K6 criteria), and 12 (1.6%) met 3 criteria. Among participants who met probable PTSD criteria (n = 31), 26 (83.9%) met any of the other 2 high-risk criteria with 24 (77.4%) of probable depression and 14 (45.2%) of high general psychological distress. Among participants who met depression criteria (n = 96), 52 (54.1%) met any of other 2 high-risk criteria with 24 (25.0%) of probable PTSD and 40 (41.7%) of high general psychological distress. Among participants who met high general psychological distress criteria (n = 58), 72.4% met any of the other 2 high-risk criteria with 40 (69.0%) of probable depression and 14 (24.1%) of probable PTSD.

Table 2 shows the demographic characteristics and the risk factors experienced by participants according to the number of high-risk criteria they met. The residual analysis revealed that participants who did not meet any high-risk criteria experienced fewer disaster-related personal (except for dead or missing family member(s)) and workplace factors compared to those who met high-risk criteria.

**Table 2:** Risk factors experienced by participants according to the number of high-risk criteria they met.

	Yes	No	χ^2^	P
**Disaster-related personal factors**				
Near-death experience			22.65	<0.001
Meeting no high-risk criteria	377	356^a^		
Meeting 1 high-risk criterion	57^a^	8		
Meeting 2 high-risk criteria	30	12		
Meeting 3 high-risk criteria	11^a^	1		
Dead or missing family member(s)			14.20	0.003
Meeting no high-risk criteria	47	686		
Meeting 1 high-risk criterion	4	61		
Meeting 2 high-risk criteria	7^a^	35		
Meeting 3 high-risk criteria	4^a^	8		
Displacement			17.01	0.001
Meeting no high-risk criteria	150	583^a^		
Meeting 1 high-risk criterion	18	47		
Meeting 2 high-risk criteria	14	28		
Meeting 3 high-risk criteria	9^a^	3		
**Workplace factors**				
Criticism by community people			24.32	<0.001
Meeting no high-risk criteria	164	450^a^		
Meeting 1 high-risk criterion	24	41		
Meeting 2 high-risk criteria	21^a^	21		
Meeting 3 high-risk criteria	9^a^	3		
Lack of rest			25.00	<0.001
Meeting no high-risk criteria	204	410^a^		
Meeting 1 high-risk criterion	35^a^	30		
Meeting 2 high-risk criteria	24^a^	18		
Meeting 3 high-risk criteria	8^a^	4		
Lack of communication			64.71	<0.001
Meeting no high-risk criteria	191	423^a^		
Meeting 1 high-risk criterion	45^a^	20		
Meeting 2 high-risk criteria	28^a^	14		
Meeting 3 high-risk criteria	10^a^	2		

^a^ Statistically significant association by adjusted residual analysis (p<0.05)

#### Factors associated with psychological symptoms

Tables 3 and 4 show the results of the bivariate and multivariate analysis of factors associated with probable PTSD, probable depression, and high general psychological distress.

**Table 3:** Bivariate analysis of factors associated with psychological symptoms.

	Probable PTSD				Probable depression				High general psychological distress			
Bivariate analysis	β	SE	OR	P	β	SE	OR	P	β	SE	OR	P
**Age**	-0.03	0.02	0.97	0.07	-0.02	0.01	0.98	0.02	-0.02	0.01	0.98	0.06
**Gender**												
Female	0.18	0.41	1.19	0.67	0.29	0.25	1.34	0.24	0.62	0.34	1.86	0.07
**Disaster-related personal factors**												
Near-death experience	1.07	0.49	2.91	0.03	1.01	0.28	2.74	<0.01	0.95	0.34	2.57	<0.01
Dead or missing family member(s)	0.98	0.47	2.66	0.04	0.70	0.32	2.01	0.03	1.04	0.35	2.82	<0.01
Displacement	1.24	0.36	3.46	<0.01	0.64	0.23	1.90	<0.01	0.58	0.27	1.78	0.04
**Workplace factors**												
Criticism by Community people	1.09	0.36	2.96	<0.01	0.77	0.22	2.16	<0.01	1.23	0.27	3.43	<0.01
Lack of rest	0.61	0.36	1.83	<0.01	1.13	0.22	3.11	<0.01	0.98	0.27	2.65	<0.01
Lack of communication	1.54	0.40	4.68	<0.01	1.74	0.24	5.72	<0.01	1.81	0.31	6.10	<0.01

SE = standard error, OR = adjusted odds ratio

**Table 4:** Multivariate analysis of factors associated with psychological symptoms.

	Probable PTSD				Probable depression				High general psychological distress			
Multivariate analysis	β	SE	OR	P	β	SE	OR	P	β	SE	OR	P
**Age**					-0.01	0.01	0.99	0.39				
**Gender**												
Female												
**Disaster-related personal factors**												
Near-death experience	0.93	0.55	2.54	0.09	0.94	0.39	2.57	0.02	0.56	0.37	1.75	0.14
Dead or missing family member(s)	0.77	0.51	2.16	0.13	0.31	0.43	1.37	0.46	0.93	0.41	2.53	0.03
Displacement	1.03	0.39	2.81	0.01	0.80	0.29	2.23	<0.01	0.12	0.31	1.13	0.71
**Workplace factors**												
Criticism by Community people	0.84	0.39	2.31	0.03	0.25	0.29	1.28	0.40	0.94	0.30	2.55	<0.01
Lack of rest					0.31	0.47	1.36	0.51	0.38	0.30	1.47	0.21
Lack of communication	1.38	0.43	3.97	<0.01	1.45	0.30	4.27	<0.01	1.54	0.34	4.65	<0.01

SE = standard error, OR = adjusted odds ratio

#### Factors associated with probable PTSD

Multivariate analysis revealed that among disaster-related factors, “displacement” was significantly associated with probable PTSD. Among the workplace factors, “criticism by community people” and “lack of communication” were significantly associated with probable PTSD.

#### Factors associated with probable depression

Multivariate analysis revealed that among disaster-related factors, “near death experience” and “displacement” were significantly associated with probable depression. Among the workplace factors, “lack of communication” was significantly associated with probable depression.

#### Factors associated with high general psychological distress

Multivariate analysis revealed that among disaster-related factors, “dead or missing colleague(s)” was significantly associated with high general psychological distress. Among the workplace factors, “criticism by community people” and “lack of communication” were significantly associated with high general psychological distress.

## Discussion

In this study, we investigated the mental health problems and associated factors of local welfare workers (i.e., SHAKYO workers), who had played a central role in supporting the social welfare domain after the GEJE, 20–22 months after the disaster.

As we hypothesized, experiencing criticism by local community people was associated with the risk of probable PTSD and high general psychological distress in SHAKYO workers. This finding is in line with Shigemura et al.’s study [18], which reported that the experience of discrimination was associated with general psychological distress and post-traumatic response in workers at the nuclear power plant after the Fukushima Daiichi nuclear disaster.

Since local social welfare workers continuously provide support directly to the survivors who are at a higher risk and living in the same affected areas, they might become easy targets of such criticism. For example, Shiraga et al. reported that some SHAKYO workers were turned away at the door when visiting residents in temporary housing, while others faced difficulties when handling complaints of residents of temporary housing [19]. Although the precise mechanism of the association of societal rejection and prolonged PTSD response has not yet been studied well, many studies have reported that social factors could be associated with recovery from traumatic symptoms. Social support is one of the strongest factors predicting recovery from PTSD [20], and the community relationship indicated by collective efficacy [21] was negatively associated with the prevalence of probable PTSD and severity of its symptoms following disasters. Thus, the experience of criticism by affected people may damage the relationship of local welfare workers with their community and further hamper recovery from traumatic experiences.

Lack of workplace communication was also a risk factor for probable PTSD, depression, and high general psychological distress in the present participants. The findings are consonant with previous studies, which demonstrated that poor workplace communication was a risk factor for developing mental health problems in local workers at 7 [3] and 14 months [2] after the GEJE. The increasing task load after the GEJE might reduce opportunities for interpersonal communications. Additionally, because some staff members, mainly those who were working as life support advisors, were newly employed after the GEJE, they might not have had sufficient opportunity to develop the necessary relationships to promote workplace communication. Workplace communication might facilitate feeling connected and positive at the workplace and may be important for recovery from mass trauma and post-traumatic growth in local workers [22].

In accordance with the previous studies [23–25], displacement was also associated with an increased risk of mental health problems, including probable PTSD and depression. Since displacement is usually associated with many primary and secondary stressors related to housing damage, loss of property, change of living conditions, and loss of social network [26,27], such stressors might have compromised the mental health of the workers. Taken together with the results of the association of near-death experience with probable depression and loss of family member(s) with high general psychological distress, the finding reminds us of the dual difficulties of the SHAKYO workers who experienced stress as both disaster survivors and as local workers. Therefore, special attention to disaster-related personal factors as well as work-related stressors should be provided to support such local workers. Additionally, although not all SHAKYO workers had such severe personal experiences, general education about the mental health of local workers after a disaster to all staff members may help increase mutual understanding and ties at the workplace.

In the present sample, many local workers experienced both personal and workplace risk factors, and secondary stressors following the disaster had a strong impact on their mental health. This finding is in line with experiences of local nurses who were living and working in post-earthquake Canterbury, New Zealand [5]. In this report, the local nurses had to cope with challenges in home and work life, which were related to secondary stressors and became harder after the disaster, not only immediately after the disaster, but also during the subsequent recovery process. Although long-running distress in home and work life and devoting themselves to supporting others may function as coping strategies to avoid dealing with the negative emotional impact of disaster [5], these may reduce their help-seeking behavior and self-care for mental health problems, and delay the recovery process. Workplace communication may be critically important for such local workers to promote a natural recovery process in their work life.

In the present study, among disaster-related personal factors, only displacement was associated with probable PTSD, while near-death experiences and dead or missing family member(s) were not. It is possible that peritraumatic psychological processes, which were not examined in the present study, might be more influential on the existence of PTSD symptoms than exposure to the traumatic event itself [28,29].

Overall, our data demonstrated that many local welfare workers suffered from mental health problems following a large-scale natural disaster for a relatively long period. The much higher prevalence of probable PTSD and depression in the SHAKYO workers compared to the 12-month prevalence of PTSD (0.7%) and depression (2.2%) [30] in the general population in Japan is consistent with a study [2] conducted in the Miyagi prefecture using the same cutoff score as the present study; the study reported a prevalence of 7.4% of probable PTSD and 19.3% of depression in local workers 14 months after the GEJE. Compared to these, the prevalence of probable PTSD and depression in the present study seems relatively low; this can be explained by the fact that the present study was conducted 6 to 8 months after the previous study, and the prevalence of PTSD and depression is expected to decrease over time after a disaster [31–33]. However, this speculation should be interpreted cautiously, because we did not directly compare them. The rate of high general psychological distress was also greater than that in the general population (15–64 years of age) of the Miyagi prefecture before the disaster (5.5%) [34] and is comparable with rates reported among disaster survivors of the GEJE in the Miyagi prefecture who lost their homes and were living in temporary housing 18 months after the disaster (9.5%) [35] and those who were living in private rental temporary housing 21 months after the disaster (8.0%) [35]. This result implies that psychological distress among SHAKYO workers increased after the GEJE and remained high even after more than 1.5 years had passed and was similar to that of other survivors who were displaced and lived in temporary housing.

Our analysis of data provided by the participants who completed 3 symptom measures revealed that more than half of the participants with probable PTSD and depression did not meet high general psychological distress criteria. This finding casts doubt on the validity of the K6 with a total cutoff score of 13, which has been widely used in Japan after the GEJE, to screen for high-risk mental conditions including depression and PTSD. The analysis also demonstrated that participants who did not have any high-risk condition had fewer risk factors compared to those who had high-risk conditions, in accordance with the logistic regression results and thus indicates factors that may be associated with resilience or recovery processes.

The following limitations should be considered for the current study: First, the statistical power of the sample sizes may have been insufficient to demonstrate with certainty what factors influenced psychological symptoms (in particular PTSD symptoms). Therefore, the results need to be interpreted cautiously, and replication through further research is required. Second, pre-earthquake data were not available on the prevalence of high general psychological distress, depressive symptoms, or PTSD symptoms. Therefore, it is not possible to deduce from this study alone if these symptoms increased after the disaster. In addition, there was no control group, so it could not be demonstrated if the prevalence of psychological symptoms was higher in welfare workers in the affected areas than in welfare workers in other areas. To compensate for this limitation, longitudinal studies are needed to observe how psychological symptoms change over time. Third, this study used a self-administered questionnaire rather than interviews to evaluate psychological symptoms. Self-administered questionnaires, especially ones for screening, may over-identify individuals as high risk [36]. Thus, when interpreting the current results, one needs to keep in mind that the prevalence rates for high-risk individuals were not based on clinical diagnoses. However, the self-administered questionnaires used in this study have often been used in other studies, thus enabling comparison with their results. Fourth, the study was implemented through worksites, so workers who had resigned between the time of the disaster and the time of the study were not included as participants. When interpreting the results, one needs to consider that workers who had resigned in this period might have been suffering from severe psychological symptoms. Finally, we could not access the information of workers who did not participate in the workplace health examination, and there is a possibility that their characteristics were different from the participants in this study.

## Conclusion

The present study demonstrated that many local social welfare workers suffered from mental health problems 20–22 months after the GEJE. Work-related social factors including criticism by community people and lack of workplace communication were associated with the risk of post-disaster mental health problems. Therefore, special measures such as developing support systems for the local workers and promoting communication at the workplace should be considered to prevent and treat mental health problems of the local service providers.

## Supporting information

S1 Table Participant characteristics and psychological symptoms. (XLSX)

S2 Table Risk factors experienced by participants according to the number of high-risk criteria they met. (XLSX)

S3 Table Bivariate analysis of factors associated with psychological symptoms. (XLSX)

S4 Table Multivariate analysis of factors associated with psychological symptoms. (XLSX)

S1 Questionnaire J Questionnaire in Japanese. (XLSX)

S1 Questionnaire E Questionnaire in English. (XLSX)
